# Modeling the Potential Distribution of *Typha domingensis* (Pers.) in Niger Under Current and Future Climate Scenarios

**DOI:** 10.1002/ece3.72265

**Published:** 2025-10-08

**Authors:** Bourahima Adamou Moumouni, Bachirou Seyni Bodo, Manssour Abdou Maman, Aboubacar Awaïss

**Affiliations:** ^1^ Department of Soil Sciences, Faculty of Agronomy Abdou Moumouni University of Niamey Niamey Niger; ^2^ Department of Environmental Sciences, Faculty of Agricultural Sciences Boubakar Bâ University of Tillabéri Tillabéri Niger; ^3^ Department of Rural Engineering and Water and Forestry, Faculty of Agronomy Abdou Moumouni University of Niamey Niamey Niger

**Keywords:** distribution, habitat, modeling, Niger, *Typha domingensis*

## Abstract

The invasion of water bodies by *Typha domingensis* is one of the main obstacles to the management and development of wetlands. To ensure the continuous provision of services and the maintenance of development activities in these areas, it is necessary to understand the current distribution of this species, as well as its future distribution in the face of climate change. It is in this context that this study has set the following objectives: (i) identifying the explanatory variables that influence the distribution of *T. domingensis* habitat and (ii) determining its current and future distribution area in Niger. To do this, bioclimatic variables from Worldclim, slope, elevation, and 378 spatially filtered to minimize clustering *T. domingensis* occurrence points were used for scientific precision. These data were loaded into the maximum entropy‐based geographic distribution modeling program called “MaxEnt, version 3.4.4”. The area under the curve (AUC) value and the Jackknife test were used to assess the model stability and the importance of environmental variables for predictive modeling, respectively. The model yielded a high AUC value of 0.98, indicating strong predictive performance. Isothermality (76.7%), precipitation seasonality (10.4%), and altitude (6.4%) are the environmental variables that contribute most to the distribution of *T. domingensis* range. The distribution of the *T. domingensis* range currently extends along the Sahelian and Sahelo‐Sudanian agroecological zones, with a surface area of 32,036 km^2^ compared with 4930 km^2^ in future projections, representing a contraction of around 85%. The approach could be promising for predicting the potential distribution of invasive aquatic plant species and can therefore be an effective tool for adapting conservation and management policies for affected wetlands.

## Graphical abstract

The MaxEnt modeling program revealed that isothermicity, seasonal rainfall, and altitude are the environmental variables that contribute most to the distribution of the range of *Typha domingensis* . This range currently extends along the Sahelian and Sahelo‐Sudanese agroecological zones, covering an area of 32,036 km^2^, compared to 4930 km^2^ in future projections, representing a reduction of nearly 85% of its habitat. This approach could prove promising for predicting the potential distribution of invasive aquatic plant species and may therefore be an effective tool for adapting conservation and management policies for affected wetlands.

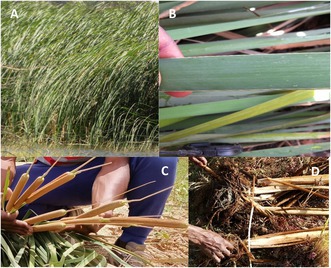

## 1 Introduction

**FIGURE 1:**
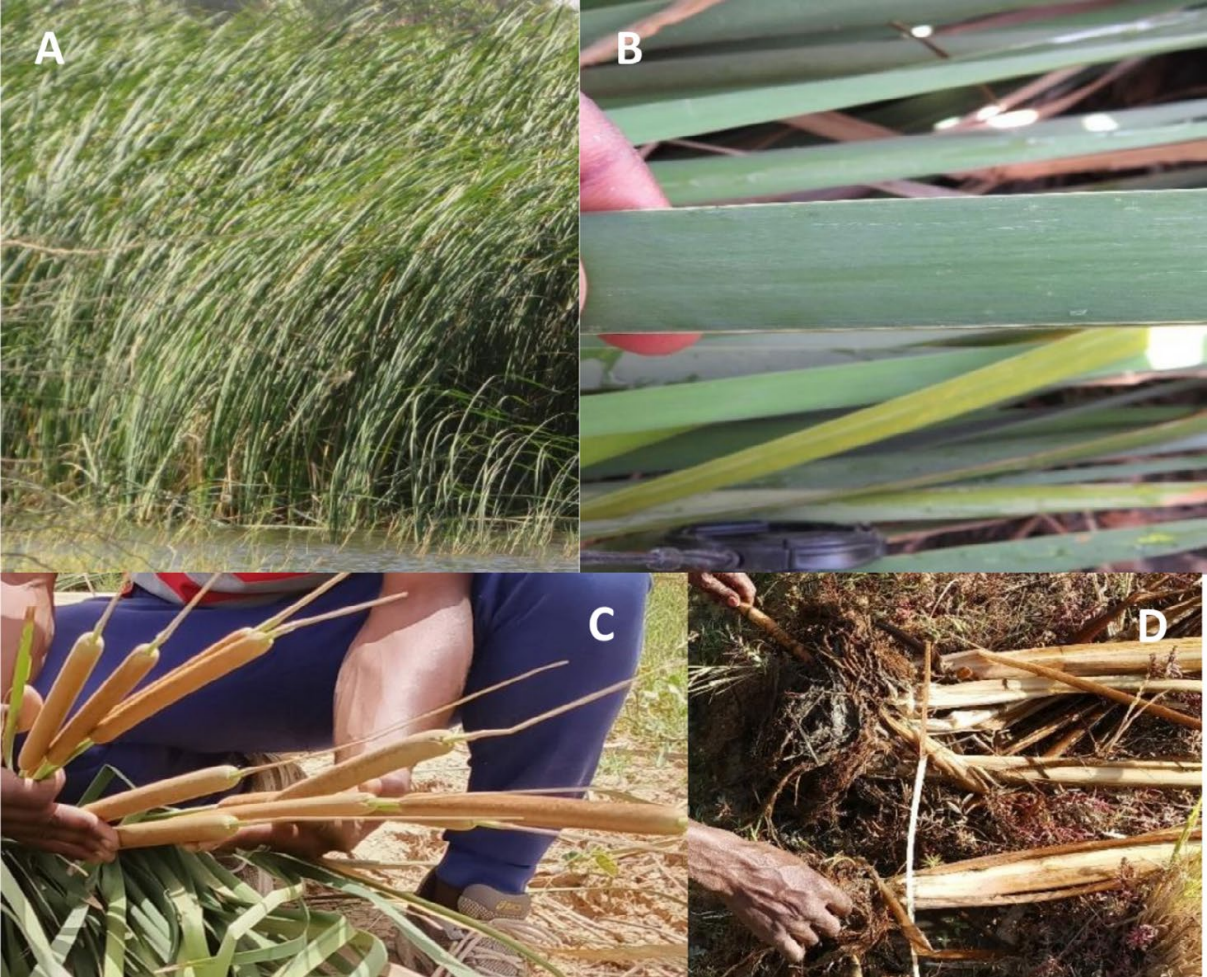
View of *Typha domingensis* : (A) tuft; (B) leaf; (C) ears; (D) roots.

The increase in temperatures highlighted in the Fifth Assessment Report of the Intergovernmental Panel on Climate Change (IPCC) poses a serious threat to the sustainability of global ecosystems (Dawson 2011). With global warming, species distribution, population size, and genetic diversity will change (Bertrand et al. 2011). Among the species, invasive aquatic plants that establish themselves in wetland ecosystems, such as *Typha domingensis* , lead to their general dysfunction (Pimentel et al. 2001) and a loss of local biodiversity (Richard et al. 2000; Brooks et al. 2004; Boers et al. 2007; Magnnon et al. 2007). The species produces numerous offspring, is itself fertile, and has a strong potential for expansion over large areas (Heger and Trepl 2003). It also tolerates large variations in water level (Wilcox and Xie 2008) and preferentially occupies eutrophic (Woo and Zedler 2002), stagnant, or even anaerobic environments (Figure 1). The species' mode of reproduction, based on seeds and vegetative propagation (Diagne et al. 2010), makes its spread rapid and difficult to control. According to a study conducted by the Integrated Management of Proliferating Aquatic Plants Project (PGIPAP) in 2009 (Amani and Barmo 2010) on 24 ponds in Niger, 70.7% of the total area is invaded by *T. domingensis* . The fight against *T. domingensis* has been on the agenda of management and science for many decades (Wilcox et al. 2017) through the development of certain control methods. These include fire control, chemical control, and manual control (Hellsten et al. 1999; Dancette and Sarr 2002; Theuerkorn and Henning 2005; Johnson et al. 2019). But it is clear that the process of invasion of wetlands by *T. domingensis* continues in Niger because the almost permanent humidity of the environment limits the fight against fire, while chemical control poses health and environmental problems. Mowing, the most widely used method in Niger, remains tedious due to the vastness of the areas to be treated. Preventing the proliferation of the species and its spread to other wetlands in the country, which have not yet been affected, is therefore by far the most cost‐effective form of management. This requires knowledge of its current and future distribution area, in the face of climate change. This is why this study sets the following objectives: (i) to identify the environmental variables that influence the spatial distribution of *T. domingensis* and (ii) to determine the current and future distribution area of the species in Niger.

## 2 Materials and Methods

### 2.1 Study Area

**FIGURE 2:**
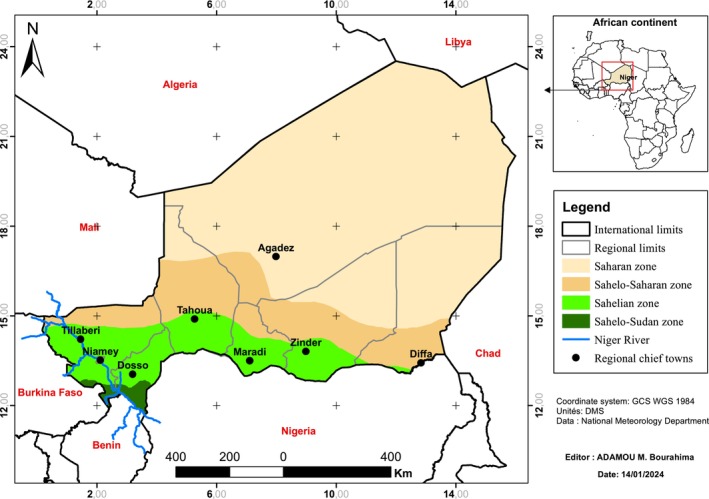
Location map of the Republic of Niger.

This study covered the entire territory of Niger, which lies between the parallels 11.61° and 23.55° North and the meridians 16° and 0.16° East (Figure 2). The country covers an area of 1,266,491 km^2^ (formerly 1,267,000 km^2^) with a particularly arid climate, belonging to one of the hottest areas on planet Earth. There are two types of hot climates: a desert climate over most of its area (North of the country), and a tropical climate with a single rainy season (South of the country). In this tropical zone, there is a so‐called cold season (November to February) characterized by average temperatures of 10°C, a dry and hot season (March to June) with temperatures of around 45°C, and a rainy season (July to September) characterized by an average temperature of 33°C. Rainfall is characterized by an annual rainfall of < 150 mm in the Saharan zone. In the Sahel‐Saharan zone, it is between 150 and 300 mm. It remains between 300 and 600 mm and fluctuates between 600 and 750 mm respectively in the Sahelian zone and the Sahel‐Sudanian zone (SE/CNEDD 2006; Van Vyve 2006). Six types of soils dominate the territory of Niger (FAO 2000) and are generally sandy in texture. Of these soils, vertisols and hydromorphic soils are the most common in areas invaded by *T. domingensis* .

### 2.2 Methodological Approach

Modeling the potential distribution of a species' habitat requires knowledge of the environmental conditions that are suitable for it. The importance of climate in explaining plant distribution was highlighted very early on by De Candolle (1855) and Franklin and Miller (2010). It is therefore established that modeling the distribution of a species is generally more effective when the environmental variables used, likely to condition its presence, are climatic and/or eco‐physiological parameters (Austin 2007). In this study, the occurrence sites of *T. domingensis* were used as sampling points, and the rasters of bioclimatic, slope, and altitude variables covering the whole of Niger were used as characteristics of the environmental conditions of the species. The modeling program called “MaxEnt, version 3.4.3” was chosen because of its good capacity to operate with only presence data (Phillips and Dudík 2008) across various modeling scenarios (Elith and Leathwick 2009) and even in contexts of correlated variables (Elith et al. 2011).

#### 2.2.1 Species Occurrence Data

The coordinates of the *T. domingensis* occurrence locations were collected using a GPS (with ±5 m accuracy) during field campaigns covering the period “May 2023 to January 2024.” Given the difficult access to the species, which is most often found in water, the coordinates were taken close to the species' clumps, and Google Earth was then used to position them in the clumps. To reduce bias caused by spatial aggregation, avoid model adjustment, and prevent model performance from being exaggerated, filtering was carried out at a spatial thinning distance of around 16,800 m using ArcGis 10.8 software. Thus, out of a total of 767 occurrences recorded, 378 were selected and converted into CSV files for modeling.

#### 2.2.2 Environmental Variables

According to Guisan and Zimmermann (2000), temperature and precipitation are the environmental variables that most affect species distribution when modeling over a large area. Based on this information, 21 environmental variables were used to build the model, including 19 bioclimatic variables (Table 1), an elevation layer, and a slope layer. The bioclimatic variables and the elevation layer were all collected at a resolution of 30 arc‐seconds (Hijmans et al. 2005) from WorldClim (Fick and Hijmans 2017). The slope layer was obtained by performing a slope calculation in ArcGis using the elevation layer. These variables were subjected to a multicollinearity test using Pearson correlation analysis in ArcGis (Table 2). When the cross‐correlation coefficient was > ±0.8, the corresponding variables were excluded. Of the 21 initial variables, only nine were selected and converted to ASCII format for model building (Table 1). For the projections of *T. domingensis* habitat distribution under future climate conditions, the new series of climate scenarios “Shared Socioeconomic Pathways” (SSP) was retained, and the SSP245 scenario (called realistic), for the period 2021–2040, was chosen. The data were subsequently extracted under the MIROC6 model, which gave good climate prediction results in the study by Deme et al. (2017) on “Rural societies facing climate and environmental changes in West Africa”.

**TABLE 1:** Environmental variables.

Code	Environmental variables	Unit
bio_1	Annual mean temperature	°C
bio_2	Mean diurnal range (mean of monthly (max temp − min temp))	°C
bio_3	Isothermality (BIO2/BIO7) (×100)	%
bio_4	Temperature seasonality (standard deviation × 100)	°C
bio_5	Max temperature of warmest month	°C
bio_6	Min temperature of coldest month	°C
bio_7	Temperature annual range (BIO5–BIO6)	°C
bio_8	Mean temperature of wettest quarter	°C
bio_9	Mean temperature of driest quarter	°C
bio_10	Mean temperature of warmest quarter	°C
bio_11	Mean temperature of coldest quarter	°C
bio_12	Annual precipitation	mm
bio_13	Precipitation of wettest month	mm
bio_14	Precipitation of driest month	mm
bio_15	Precipitation seasonality (coefficient of variation)	%
bio_16	Precipitation of wettest quarter	mm
bio_17	Precipitation of driest quarter	mm
bio_18	Precipitation of warmest quarter	mm
bio_19	Precipitation of coldest quarter	mm
Ele	Elevation	m
Slo	Slope	°

**TABLE 2:** Pearson correlation between environmental variables.

Layer	Bio_1	Bio_2	Bio_3	Bio_4	Bio_5	Bio_6	Bio_7	Bio_8	Bio_9	Bio_10	Bio_11	Bio_12	Bio_13	Bio_14	Bo_15	Bio_16	Bio_17	Bio_18	Bio_19	Ele	Slo
Bio_1	1																				
**Bio_2**	**−0.38**	1																			
**Bio_3**	**0.75**	**−0.33**	1																		
Bio_4	−0.78	0.62	−0.93	1																	
**Bio_5**	**0.36**	**0.53**	**−0.07**	**0.23**	1																
Bio_6	0.88	−0.65	0.89	−0.97	−0.07	1															
Bio_7	−0.73	0.76	−0.86	0.97	0.33	−0.96	1														
Bio_8	−0.34	0.62	−0.74	0.83	0.62	−0.71	0.83	1													
Bio_9	0.83	−0.53	0.86	−0.91	−0.03	0.92	−0.88	−0.68	1												
Bio_10	0.5	0.16	−0.09	0.12	0.88	0.09	0.14	0.56	0.07	1											
Bio_11	0.91	−0.56	0.91	−0.96	0.007	0.99	−0.93	−0.67	0.92	0.14	1										
Bio_12	0.62	−0.62	0.82	−0.88	−0.27	0.86	−0.88	−0.8	0.76	−0.13	0.85	1									
Bio_13	0.67	−0.63	0.86	−0.93	−0.27	0.9	−0.92	−0.83	0.81	−0.14	0.89	0.98	1								
**Bio_14**	**−0.09**	**−0.02**	**−0.06**	**0.04**	**−0.08**	**−0.06**	**0.03**	**−0.04**	**−0.06**	**−0.07**	**−0.07**	**−0.02**	**−0.03**	1							
**Bo_15**	**0.7**	**−0.32**	**0.76**	**−0.79**	**−0.08**	**0.75**	**−0.73**	**−0.58**	**0.77**	**−0.08**	**0.76**	**0.48**	**0.58**	**−0.08**	1						
Bio_16	0.64	−0.63	0.84	−0.91	−0.27	0.88	−0.9	−0.82	0.79	−0.14	0.87	0.99	0.99	−0.02	0.53	1					
**Bio_17**	**−0.48**	**−0.05**	**−0.29**	**0.24**	**−0.37**	**−0.31**	**0.19**	**−0.09**	**−0.22**	**−0.38**	**−0.35**	**−0.1**	**−0.14**	**0.34**	**−0.39**	**−0.12**	1				
Bio_18	0.53	−0.47	0.68	−0.7	−0.18	0.7	−0.71	−0.6	0.59	−0.06	0.7	0.84	0.81	−0.02	0.36	0.82	−0.11	1			
**Bio_19**	**−0.06**	**−0.02**	**−0.04**	**0.02**	**−0.05**	**−0.03**	**0.01**	**−0.02**	**−0.03**	**−0.04**	**−0.04**	**0.01**	**−0.002**	**0.03**	**−0.06**	**0.002**	**0.14**	**−0.003**	1		
**Ele**	**−0.79**	**0.09**	**−0.69**	**0.6**	**−0.4**	**−0.66**	**0.52**	**0.26**	**−0.67**	**−0.41**	**−0.72**	**−0.52**	**−0.55**	**0.11**	**−0.54**	**−0.53**	**0.49**	**−0.4**	**0.07**	1	
**Slo**	**−0.24**	**−0.03**	**−0.16**	**0.12**	**−0.25**	**−0.16**	**0.08**	**0.02**	**−0.17**	**−0.24**	**−0.19**	**−0.12**	**−0.13**	**0.05**	**−0.08**	**−0.13**	**0.18**	**−0.05**	**0.02**	**0.37**	1

*Note:* Variables in bold are those used to model the habitat of *T. domingensis* .

#### 2.2.3 Construction of the Species Habitat Distribution Model

Specie occurrence data and environmental variables were introduced into MaxEnt to model current and future potential habitats of *T. domingensis* . 70% of the points of occurrence were randomly selected as training data for model construction, and the remaining 30% as test data for model evaluation. Cross‐validation was set on 10 repetitions in the repeated run type, and the Jackknife test was used to measure the contribution and importance of environmental variables in the potential distribution of the species. The logistic output format was preferred due to its significantly better performance (Phillips and Dudík 2008). The other parameters were kept as default, with a regularization multiplier of 1, a convergence threshold of 10^−5^, a maximum number of iterations of 500, and a maximum number of background points of 10,000. Finally, the average result of the 10 cross‐validation runs of the model was considered as the ultimate habitat suitability.

Manel et al. (1999) demonstrated the need to evaluate predictive models of habitat distribution. The receiver operating characteristic (ROC) curve generated by the program and the average omission rate of the test data were used to evaluate the prediction of the distribution of the model obtained. The ROC curve, which produces an area under the curve (AUC) value, is an acceptance curve in which the abscissa represents the false positive rate (1 − specificity) and the vertical axis represents the true positive rate (1 − omission rate). It reflects the compromise between sensitivity and specificity. It corresponds to the probability that a randomly selected presence site is ranked above a randomly selected absence site (Phillips and Dudík 2008). According to Swets (1988), a model is poor when its AUC value < 0.75 and good if its AUC > 0.90. For Elith (2000), a model is potentially useful when its AUC > 0.75. The omission rate varies from 0 to 1 and is used to evaluate the model's ability to detect real presences. An omission rate of 0 indicates that the model correctly detects all real presences, while a rate of 1 indicates that the model detects none.

#### 2.2.4 Determination of Potential Habitat Suitable for the Species

The output of the MaxEnt model was a probability map of the potential distribution of the species' habitat, ranging from 0 (least suitable habitat) to 1 (most suitable habitat). To delineate suitable and unsuitable habitats for the species, the threshold “Minimum training presence” was used to convert the continuous maps into binary maps. Thus, the threshold‐dependent mean value (0.18) was used to delineate the probability of habitat suitability (i.e., ≥ 0.18) and unsuitability (i.e., < 0.18). The delineation was achieved by reclassifying the continuous map, using the spatial analysis tools of ArcGIS. It was subsequently possible to assess the gain or loss in the potential distribution area of the species at the national level following climate projections (Barthélémy et al. 2019).

## 3 Results

### 3.1 Relative Contribution of Environmental Variables

**FIGURE 3:**
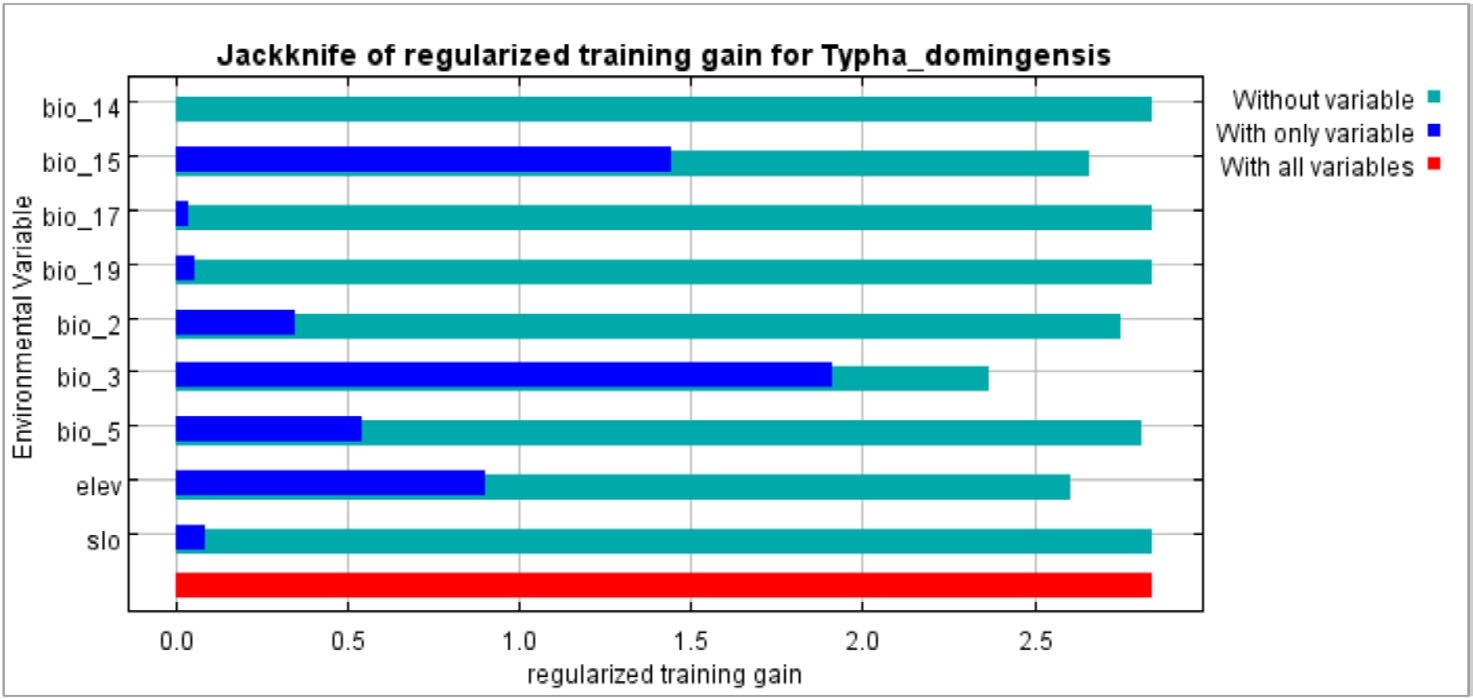
Influence of variables in predicting the habitat of *Typha domingensis*.

The MaxEnt model achieved a high AUC value of 0.98, suggesting good predictive ability. This good predictive ability is confirmed by the omission rate, which is close to zero (0.0085). The contribution of model‐fitting variables in predicting the range of *T. domingensis* is shown in Table 3. Isothermality (Bio_3) and precipitation seasonality (Bio_15) are respectively the environmental variables that contribute most. Slope, precipitation in the driest month, and precipitation in the coldest quarter provide no information. These results are consistent with the Jackknife test evaluation (Figure 3), which also indicates that Isothermality is the environmental variable that significantly increases and decreases the model accuracy depending on whether it is used in isolation or omitted. It is followed by altitude and precipitation seasonality.

**TABLE 3:** Contribution of variables in the establishment of *Typha domingensis* habitat.

Variable	Permutation importance
**bio_3**	**76.7**
bio_2	4.9
**bio_15**	**10.4**
**Ele**	**6.4**
bio_5	1.4
bio_17	0.1
Slo	0.0
bio_19	0.0
bio_14	0.0

*Note:* The values in bold highlight the variables that contributed the most.

### 3.2 Potentially Suitable Habitats of *Typha domingensis* in Niger

**FIGURE 4:**
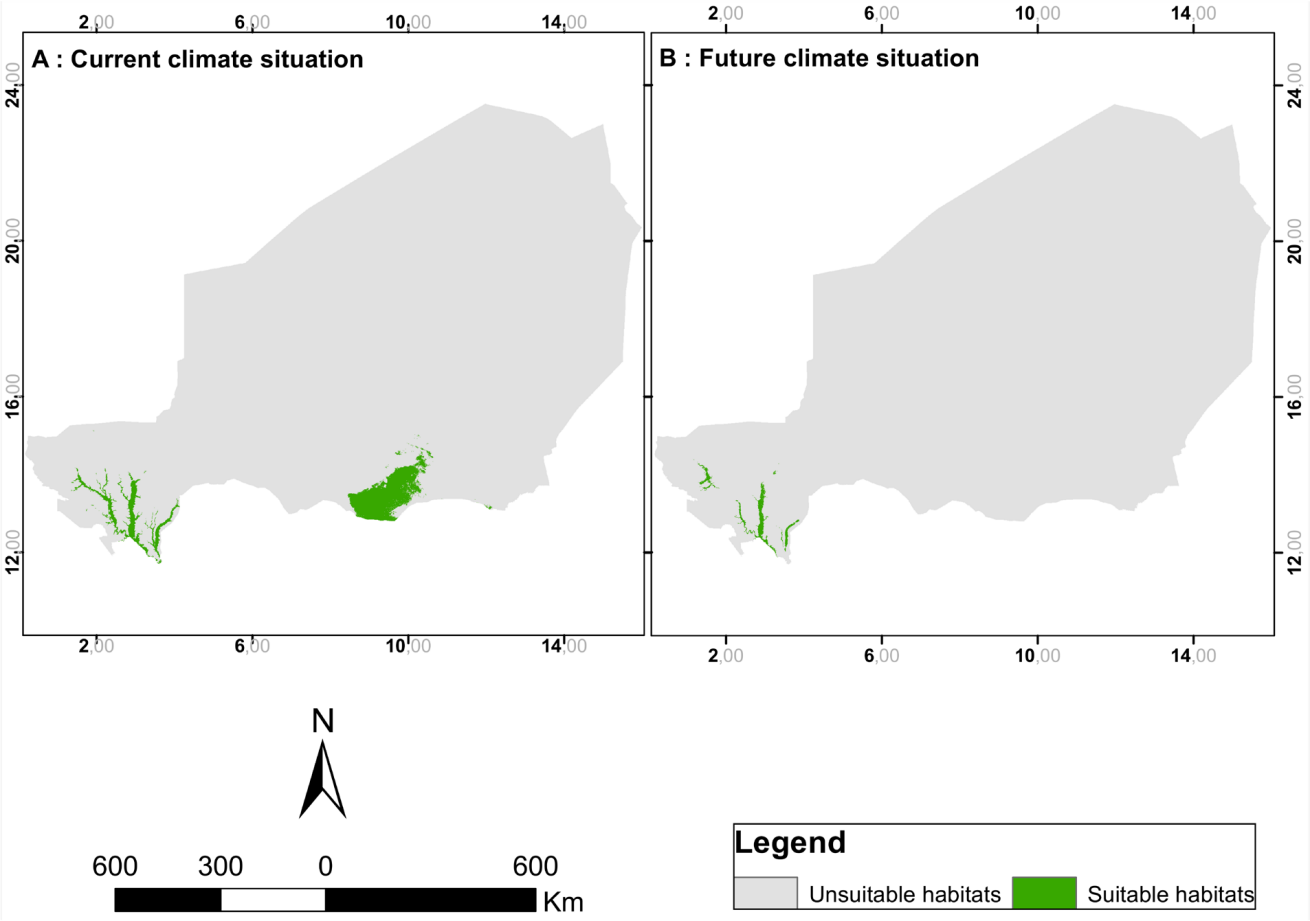
Potential distribution of *Typha domingensis* habitats in Niger.

Whether under current or future climatic conditions, the habitat of *T. domingensis* (Figure 4) is located in the Sahelian and Sahelo‐Sudanian agroecological zones of the country. In these zones, its habitat currently covers an area of 32,036 km^2^ compared to 4930 km^2^ for the future climate projection, a reduction of approximately 27,106 km^2^ (85%) compared to the current situation. This reduction is observed with the disappearance of habitats in the south‐east of the Sahelian zone.

## 4 Discussion

As demonstrated by Thorp and Lynch (2000) in Thuiller et al. (2005), the invasion potential of a species can be identified and assessed before an invasion occurs. One of the primary strengths of this modeling‐based approach is the detection of potential source areas for new invasions. This approach effectively identifies areas at high risk of *T. domingensis* invasion. It is in this approach that this work attempted to determine the potential habitat distribution of *T. domingensis* in Niger. The model developed for this purpose indicates good performance, which would be due to the number of spatially unique occurrences on the one hand and, on the other hand, to the sample size, which largely meets the minimum size (10) depending on the number of variables used (Austin et al. 2017). According to Phillips et al. (2004), the program used (MaxEnt) has the capacity to produce good results with small sample sizes. However, the distribution results obtained with a large number of occurrences turn out to be better (Hernandez et al. 2006; Wisz et al. 2008). Also, the distribution of observations in the environmental space can influence the performance of the models (Toffa et al. 2022). Indeed, depending on whether the species has a narrow distribution relative to the study area described by the environmental layers, the AUC values tend to be high (Phillips 2017). The high AUC value obtained could also be influenced by the vastness of the territory of Niger compared to the number of occurrence points used.

Cross‐referencing the results relating to the contribution of environmental variables to the model prediction clearly shows that the variables that contribute significantly to the spatial distribution of *T. domingensis* are: isothermality, precipitation seasonality, and altitude. This means that the presence of these three variables taken individually led to a strong increase in the predictive power of the model and therefore seems to provide the model with information that is not present in the other variables. The other environmental variables contributed only very little to the model prediction (Moukrim et al. 2018). Contrary to expectations that bioclimatic variables dominate at broad scales (Guisan and Zimmermann 2000; Gaudreau et al. 2015), altitude—a topographic variable—also made a significant contribution. According to Toffa et al. (2022), altitude has indirect effects on species as it acts on climatic variables. However, it is important to remain vigilant since variables such as soil types, land use, and anthropogenic factors were not taken into account in this study. These variables can interact with climatic variables (Stanton et al. 2012), and their inclusion as categorical variables negatively affects the models (Toffa et al. 2022). These results, however, allow us to consider these environmental variables as an important lever influencing the spatial distribution of *T. domingensis* in Niger.

The distribution of *T. domingensis* habitat observed in the Sehelian and Sahelo‐Sudanian zones could be due to the fact that these are the best‐watered areas of the country, with an average rainfall exceeding 300 mm per year. These areas also contain the low‐lying wetlands. This result differs from many studies that have shown that species are moving to higher altitudes or latitudes in response to global warming (Chen et al. 2011). Furthermore, the resolution of the environmental variables used may not correspond to the actual ecological resolution of *T. domingensis* , which may be finer. This would mask small areas that could potentially be colonized. Although Deme et al. (2017) predicted an increase in rainfall during the period 2011–2040 in the Sahel (east of 10° W) and a warming greater than 2°C (north of 12.5° N), a reduction in the species' habitat is expected. Given the variables that influence the distribution of the species, the predicted increase in rainfall is expected to lead to an expansion of the species' habitat, but the rise in temperatures could explain its decline. Temperature stability would therefore be a determining factor in the evolution of the species' habitat. Furthermore, this result could be due to uncertainties related to the measurement of climate data. In fact, the failure to include biotic factors in the models, particularly species plasticity, limits the robustness of the results of projections of the impact of climate change on species distribution (Toffa et al. 2022). However, this allows us to predict a reduction in the species' habitat due to the rise in temperature.

## 5 Conclusion

As an invasive species of wetlands, knowledge of the potential habitat of *T. domingensis* appears to be one of the effective and efficient means of prevention but also, and above all, of control and management of these environments of proven socioeconomic and environmental utility. Predictive models enable managers to forecast and anticipate potential changes in these sensitive ecosystems. This allows them to make more informed decisions and develop effective management strategies to protect and/or restore these precious ecosystems. The model developed for this purpose has proven to be robust, with an AUC value bordering on excellence. Climatic variables (precipitation and temperature) and altitude are the most significant variables in this distribution. The distribution map of the current and future habitat of *T. domingensis* shows the species established along the Sahelian and Sahelo‐Sudanian agroecological zones, with a reduction in its range in the future climate projection.

## Author Contributions

**Bourahima Adamou Moumouni:** conceptualization (lead), data curation (equal), formal analysis (lead), methodology (equal), writing – original draft (equal), writing – review and editing (lead). **Bachirou Seyni Bodo:** investigation (equal), methodology (equal), project administration (equal), supervision (lead), validation (equal), visualization (equal). **Manssour Abdou Maman:** investigation (equal), project administration (equal), resources (lead), supervision (equal), visualization (equal). **Aboubacar Awaïss:** supervision (equal), validation (equal), visualization (equal).

## Conflicts of Interest

The authors declare no conflicts of interest.

## Data Availability

The data supporting the conclusions of this study can be obtained as follows: environmental variables at https://www.worldclim.org/data/worldclim21.html and *Typha domingensis* occurrences on Zenodo via the following link: https://zenodo.org/records/16785488. The variables are rasters, and the occurrences are in an Excel file.
